# Ferroptosis-related mechanisms in prion diseases provide insights into neurodegeneration and reveal therapeutic implications

**DOI:** 10.1016/j.redox.2026.104155

**Published:** 2026-04-04

**Authors:** Mohammed Zayed, Hilal Tayara, Byung-Hoon Jeong

**Affiliations:** a Korea Zoonosis Research Institute, Jeonbuk National University, Iksan, 54531, Republic of Korea; b Department of Bioactive Material Sciences, Jeonbuk National University, Jeonju, 54896, Republic of Korea; c Department of Surgery, College of Veterinary Medicine, Qena University, Qena, 83523, Egypt; d School of International Engineering and Science, Jeonbuk National University, Jeonju, 54896, Republic of Korea

**Keywords:** Cell death, Ferroptosis, Neurodegenerative diseases, Pathogenesis, Prion disease, Therapeutics

## Abstract

Prion diseases are a group of fatal neurodegenerative disorders caused by misfolded proteins. Understanding the regulatory networks of ferroptosis in prion diseases could unveil new diagnostic and therapeutic strategies. To explore this, we systematically evaluated ferroptosis-associated alterations across human sporadic Creutzfeldt–Jakob disease (sCJD) brain samples, the ME7-infected mouse model, and *in vitro* using PrP^106-126^-treated SH-SY5Y cells. In sCJD patients, we observed a significant decrease in GPX4 expression, accompanied by elevated lipid peroxidation, as confirmed by malondialdehyde assays. Furthermore, *in vitro* experiments using PrP^106-126^-treated cells confirmed that ferroptosis-related mechanisms actively contribute to cell death, characterized by elevated lipid peroxidation, reactive oxygen species, and increased intracellular Fe^2+^ levels, as well as diminished glutathione activity. Critically, pharmacological inhibition with ferrostatin-1 effectively mitigated this neurotoxicity, consistent with a ferroptosis-related mechanism. To validate these findings *in vivo*, we demonstrated that ME7-infected mice exhibited significantly lower levels of GPX4 and SLC7A11, which correlated with increased 4-hydroxynonenal and neuronal damage. Finally, bioinformatic analysis of the GSE124571 dataset identified a distinct transcriptomic signature of 130 differentially expressed ferroptosis-related genes in sCJD patients. These results collectively suggest that ferroptosis-associated alterations are involved in prion-associated neurodegeneration, offering valuable pathophysiological insights into disease progression.

## Highlights

• Ferroptosis-related stress markers are observed in human sCJD and ME7 mouse brains.
• GPX4 depletion and lipid peroxidation mark the pathological landscape of sCJD.
• Ferrostatin-1 rescues PrP^106-126^-induced neurotoxicity and oxidative stress *in vitro*.
• Reduced GPX4 expression correlates with neuronal damage in ME7-infected mice.
• Transcriptomics reveals dysregulation of ferroptosis-related pathways in sCJD.

## 1 Introduction

Prion diseases, also known as transmissible spongiform encephalopathies (TSEs), are a category of deadly neurodegenerative conditions that result from the accumulation of misfolded prion protein (PrP^Sc^), which triggers a cascade of pathogenic processes in the brain [1]. Despite advances in understanding these diseases, the precise mechanisms leading to neurodegenerative outcomes remain elusive, posing a significant barrier to the development of effective therapies [2,3]. It is well known that the conversion of normal prion protein (PrP^C^) into PrP^Sc^, a β-sheet-rich form, is a crucial process in the development of prion diseases. However, the exact mechanism behind this conversion remains unknown.

In sporadic Creutzfeldt-Jakob disease (sCJD) patients, elevated levels of oxidative stress and lipid peroxidation have been consistently observed, suggesting that these processes are initial pathogenic events rather than terminal byproducts [[4], [5], [6], [7]]. In addition, lipid peroxidation is associated with prion disease, which may represent a pathogenic event in disease progression [8]. Studies involving sCJD patients and prion strain-infected animal models have reported various forms of neuronal cell death, including apoptosis, necroptosis, and autophagy [9,10]. Ferroptosis, a distinct form of programmed cell death, is characterized by iron-dependent lipid peroxidation [11]. It has gained considerable attention in recent years due to its involvement in various diseases, including neurodegenerative diseases [12,13]. Therefore, understanding the mechanistic role of ferroptosis in prion diseases could serve as a diagnostic biomarker and promote the development of therapeutic strategies to mitigate these disorders. Further research is essential to elucidate the fundamental mechanisms underlying prion diseases and their neurodegenerative processes.

A key feature of ferroptosis is its reliance on iron and lipid metabolism [14]. Unlike other forms of cell death, such as apoptosis, necrosis, and autophagy, ferroptosis is triggered by the accumulation of lipid peroxides produced by the oxidative degradation of lipids [14]. The inability of cells to neutralize these lipid peroxides is primarily due to the depletion of antioxidants, such as glutathione (GSH), and the inactivation of glutathione peroxidase 4 (GPX4), ultimately leading to ferroptotic cell death [15]. Consequently, lipid peroxidation plays a central regulatory role in cell damage mediated by ferroptosis [16]. In contrast, the dysregulation of iron metabolism can have severe consequences for neuronal health and survival, significantly impacting brain function [17]. In prion diseases, dysregulation of iron homeostasis and oxidative stress are prominent pathological features [18], suggesting a potential relation between prion disease pathogenesis and ferroptosis. Previous studies have reported imbalances in iron homeostasis in mice and hamster models infected with scrapie [19,20]. For instance, Kim et al. demonstrated that the brains of scrapie-infected mice exhibit significantly increased expression levels of key proteins involved in iron metabolism compared to non-infected controls [21]. Research has shown that brains infected with prions exhibit markedly increased iron levels, which facilitate the generation of reactive oxygen species (ROS) through the Fenton reaction [22]. These ROS initiate lipid peroxidation, leading to neuronal cell damage and death [23]. The results suggest that elevated levels of ROS in the brain, combined with increased lipid peroxidation and lower levels of GSH, may contribute to the progression of neurodegenerative diseases [24]. These observations highlight a critical need to rigorously validate the ferroptotic pathway within the context of human pathology and to determine its functional contribution to neurodegeneration.

In the present study, we first investigated whether the biochemical hallmarks of ferroptosis are present in the brain tissue of human sCJD patients compared with those of normal controls. To further extend and support these human findings, we used the ME7-infected mouse model to explore ferroptosis progression *in vivo*. We used PrP^106-126^-treated SH-SY5Y cells to evaluate the neuroprotective potential of the ferroptosis inhibitor ferrostatin-1 (Fer-1). Finally, we used transcriptomic data from the Gene Expression Omnibus (GEO) to define a broad ferroptotic gene signature in sCJD, providing a comprehensive cross-species validation of ferroptosis as a contributor to prion-induced neurodegeneration.

## 2 Materials and methods

### 2.1 Human brain tissue homogenate analysis

Human post-mortem brain samples from sCJD patients and matched controls were obtained from the University of Edinburgh (detailed patient information in Table S1). All samples were obtained with informed consent under institutional review board (IRB)-approved protocols. All procedures were approved by the IRB of Jeonbuk National University and followed the 1964 Declaration of Helsinki and its later amendments or comparable ethical standards (approval number: 2020-10-014). Brain tissue homogenates were prepared in RIPA buffer (Thermo Fisher Scientific, 89901**)** containing protease inhibitors (Sigma-Aldrich, P8465) for Western blot analysis of GPX4 protein levels (see Western Blot Methods section). In addition, malondialdehyde (MDA), which is a product of membrane lipid peroxidation that is positively associated with ferroptosis, was assessed in the protein lysate of the brain tissue (see Evaluation of MDA levels section).

### 2.2 Reagents and treatment of SH-SY5Y cells

PrP^106-126^ (KTNMKHMAGAAAAGAVVGGLG; >95 % purity) was synthesized by Peptron (Yuseong-gu, Daejeon, South Korea). The peptide was dissolved in dimethyl sulfoxide (DMSO) (Biosesang, 67-68-5) at a concentration of 10 mM and stored at −80 °C. SH-SY5Y cells were obtained from the Korean cell line bank (Seoul, Korea) and cultured in DMEM/Nutrient Mixture F-12 (DMEM-F12; Gibco, 11320033) supplemented with 10 % FBS (Sigma-Aldrich, F2442**)** at 37 °C in a humidified incubator with 5 % CO_2_. In our previous study, the optimized condition for PrP^106-126^ treatment was established, demonstrating that a concentration of 100 μM for 24 h significantly induced neurotoxicity [25]. Erastin (Selleckchem, S7242) at a concentration of 10 μM and Fer-1 (Sigma-Aldrich, SML0583) at a concentration of 2 μM were used according to previous studies [[26], [27], [28]]. SH-SY5Y cells were treated with media containing DMSO (control), PrP^106-126^ (100 μM), erastin (10 μM), PrP^106-126^ with Fer-1, and erastin with Fer-1 or Fer-1 (2 μM) for 24 h at 37 °C with 5 % CO_2_.

### 2.3 Measurement of cell viability and release of lactate dehydrogenase (LDH)

SH-SY5Y cells (1 × 10^4^ cells) were plated in a 96-well plate for 24 h. Cells were treated as described above for 24 h at 37 °C and 5 % CO_2_. A Cell Counting Kit-8 assay kit (CCK-8, Sigma-Aldrich, 96992) was used to determine cell viability according to the manufacturer's instructions. In brief, CCK-8 solution was added to each well, and the cells were incubated for 2 h. Absorbance was measured at 450 nm using a microplate reader (SpectraMax Plus 384, Molecular Devices, USA). To measure LDH release into the culture medium, the manufacturer's instructions were followed using an LDH Cytotoxicity Assay Kit (Antibodies.com, A319649). In summary, 100 μl of culture media from each treatment group was placed into a new 96-well plate. Following the addition of 100 μL of reaction solution to each well, the plate was incubated for 30 min at 37 °C. The LDH release was assessed by measuring the absorbance at 565 nm with a microplate reader (Molecular Devices).

### 2.4 Evaluation of intracellular Fe^2+^ levels

To detect intracellular Fe^2+^ levels, SH-SY5Y cells were seeded in a 24-well plate (4 × 10^4^ cells/well). Following 24 h of treatment, cells were washed with phosphate-buffered saline (PBS) (SolBio, K92683855) and then incubated with FerroOrange (1 μM, Ex: 543 nm, Em: 580 nm, Dojindo, F374-10) for 30 min at 37 °C with 5% CO_2_. The cells were washed with PBS and stained with DAPI (Invitrogen, D1306). Images were captured immediately using an Olympus IX53 inverted fluorescence microscope (Shinjuku-ku, Tokyo, Japan). The fluorescent intensity in each image was quantified using ImageJ version 1.37 (National Institutes of Health, Bethesda, MD, USA).

### 2.5 Intracellular ROS detection

To measure ROS generation, intracellular ROS content was assessed by fluorescence microscopy using H_2_DCF-DA (Invitrogen, D399). In brief, H_2_DCF-DA (10 μM) was added to phenol red-free DMEM, and the SH-SY5Y cells were incubated for 30 min at 37 °C with 5 % CO_2_. Cells were washed 3 times with PBS and then observed under a fluorescence microscope. The fluorescent intensity of each image was measured using ImageJ software.

### 2.6 Evaluation of MDA levels

The MDA levels were determined using a Lipid Peroxidation MDA Assay Kit (Antibodies.com, A319696) following the guidelines provided by the manufacturer. In brief, SH-SY5Y cells were seeded in a 6-well plate and treated as described above for 24 h. Protein lysates from cells and human brain tissue were harvested using the extraction buffer provided with the kit. A 300 μl MDA detection reaction mixture was added to 100 μl of the sample or blank solution. The mixture was incubated in a 95 °C water bath for 30 min and then cooled to RT. The tubes were centrifuged at 10,000 g for 10 min at RT, and the supernatant was obtained for the assay. The MDA content was calculated from absorbance readings at 532 nm and 600 nm obtained with a microplate reader (Molecular Devices).

### 2.7 BODIPY 581/591C11 lipid peroxidation sensor

BODIPY 581/591C11 was used to determine the lipid peroxidation of treated SH-SY5Y cells. The cells were treated with 2 μM C11-BODIPY (581/591) (Invitrogen, D3861) and incubated for an additional 30 min at 37 °C with 5 % CO_2_. The fluorescence of C11-BODIPY, corresponding to the level of lipid peroxidation, was measured using an Olympus IX53 inverted fluorescence microscope. The fluorescent intensity of each image was measured using ImageJ software.

### 2.8 Measurement of reduced GSH and oxidized glutathione (GSSG)

After treating the SH-SY5Y cells as mentioned above, the cells were lysed with ice-cold 5% (w/v) metaphosphoric acid, and the supernatant was collected after centrifugation at 10,000 g for 10 min at 4 °C. Total GSH and GSSG levels were determined by the EZ-Glutathione Assay Kit (DoGenBio, DG-GPX100) following the manufacturer's instructions, as previously demonstrated [5].

### 2.9 Reverse-transcription PCR (RT-PCR)

Total RNA from treated SH-SY5Y cells was extracted using TRIzol reagent (Invitrogen, 15596018) and quantified by a NanoDrop 2000 spectrophotometer (Thermo, Waltham, USA). Complementary cDNA was synthesized through reverse transcription using ReverTra Ace-α (Toyobo, FSK-101) according to the manufacturer's instructions. Reverse-transcribed products were amplified using the SYBR Green method using a CFX96 real-time PCR system (Bio-Rad, CA, USA) according to the manufacturer's instructions. To examine mRNA expression, real-time PCR was performed using primers specific for ferroptosis-related genes (FRGs), including mitochondrial GPX4 (mGPX4), nuclear GPX4 (nGPX4), all GPX4, and SLC7A11. The expression levels were standardized using GAPDH as an internal control. The primer sequences are listed in Table S2.

### 2.10 Ethical statements and experimental model for prion disease

C57BL/6J mice were purchased from Nara Biotech (Pyeongtaek, Korea). All efforts were made to minimize harm and reduce the number of animals used in the study. The Institute of Animal Care and Use Committee of Jeonbuk National University approved all experimental procedures (approval number: JBNU 2020-080).

The prion disease model was established as previously described [25]. In brief, 100 μl of ME7 scrapie strain from terminally ill mice (provided by Roslin Institute at The University of Edinburgh) was injected intraperitoneally into mice (n = 6). At the terminal stage of disease (approximately 210 days after post-infection), the mice were sacrificed, and their brains were collected for further analysis. For the control group, six C57BL/6J mice received an intraperitoneal injection of 100 μl of PBS. The PBS-treated mice were sacrificed at the same time point as the ME7-infected mice. Brain samples (n = 3) were collected and stored at −80 °C before Western blot analysis. For assessment of proteinase K-resistant PrP^Sc^, brain samples were treated with 40 μg/mL proteinase K for 1 h at 37 °C. The proteinase K-treated samples were then heated for 10 min at 95 °C in 5x sample buffer (GenScript, MB01015). Brain samples (n = 3) were also prepared for immunofluorescence staining.

### 2.11 Immunofluorescence analysis

Whole brains were harvested and fixed in 4% paraformaldehyde solution for 24 h. The fixed tissue was subsequently immersed in a 30% sucrose solution, embedded in cryo-embedding media and optical cutting compound, and cut on a cryostat. Following sectioning at 20 μm, the sections were exposed to 0.5% Triton X-100 (BIOSESANG, T1020) for 15 min and blocked with Power Block (BioGenex Laboratories, HK085-GP) for 1 h at room temperature (RT). The sections were incubated overnight at 4 °C in a humidity chamber with glial fibrillary acidic protein (GFAP, 1:50, Santa Cruz Biotechnology, sc-33673), GPX4 (1:50, Santa Cruz Biotechnology, sc-166570), SLC7A11 (1:100, ABclonal, A13685), 4-HNE antibody (1:50, Bioss Inc., bs-6313R), and NeuN (1:50, Abcam, ab177487) overnight in a humidity chamber at 4 °C. After washing with PBST, the sections were incubated with anti-mouse Alexa Fluor-647 (Cell Signaling Technology, 4414), anti-mouse Alexa Fluor-488 (ABclonal, A5037), anti-rabbit Alexa Fluor-488 (Cell Signaling Technology, 4412S), and anti-rabbit Alexa Fluor-594 (ABclonal, A5039) for 1 h at RT. After washing, the slides were mounted with Prolong Gold Antifade mounting media (Invitrogen, P36930) and imaged under a Zeiss Axio-Imager M2 microscope, equipped with an Axiocam 506 color camera, using the same settings. Image intensity was analyzed using ImageJ (version 1.52; imagej.nih.gov).

### 2.12 Western blot analysis

Western blot analysis was performed using 20 % brain homogenates from two groups. Proteins were separated on a 12% polyacrylamide gel and transferred to a PVDF membrane (Amersham, GE10600023) at 100 V for 90 min. The membrane was blocked with 5% skim milk (BD Difco, K02383233) for 1.5 h at RT. The following primary antibodies were used and incubated overnight at 4 °C to identify specific proteins: SAF84 antibody (1:200, Bertin, Montigny-le-Bretonneux, A03208), GPX4 (Santa Cruz Biotechnology), SLC7A11 (ABclonal), and 4-HNE (ABclonal). Subsequently, a specific secondary antibody was utilized to detect the antigens. Immunoreactive bands were visualized using the Pierce ECL kit (Thermo Fisher Scientific, 35055). Protein levels were normalized to β-actin (Santa Cruz Biotechnology, sc-47778) for quantification.

### 2.13 Data acquisition and processing

The GSE124571 dataset was retrieved from the GEO database (https://www.ncbi.nlm.nih.gov/geo/). This dataset investigated gene expression profiles of patients with sCJD and non-neurological controls (CTL) without a history of neurological or psychiatric disease, using microarray analysis. Total RNA was extracted from samples using the RNeasy tissue lipid kit (Qiagen), and the concentration and purity were assessed by a spectrophotometer (NanoDrop ND100, Thermo Fisher Scientific). RNA expression profiling was performed with the Illumina whole-genome HumanRef8 v2 BeadChip (Illumina, CA, USA). FRGs (n = 1085) used in this study were obtained from FerrDb database (http://www.zhounan.org/ferrdb/current/) (Accessed on 2024.12.05).

### 2.14 Analysis of differential expressions and identification of key FRGs in sCJD patients

The R package Limma (version 3.66.0) was used to assess differential gene expression. Genes meeting the criteria of an adjusted P-value <0.05 and an absolute log2 fold change (FC) > 1 were classified as differentially expressed genes (DEGs). A heatmap was generated using a heatmap package to visualize the top 100 FRGs and their expression patterns between the sCJD and control (CTL) groups. Functional enrichment analysis, including Gene Ontology (GO) and Kyoto Encyclopedia of Genes and Genomes (KEGG) pathway analysis, was conducted using the ClusterProfiler package in R (version 4.8.3).

### 2.15 Validation for RNA-seq results

RT-PCR was performed to evaluate the expression levels of the selected genes and to validate the RNA-seq results for DDIT4, NQO1, and HMOX1, as described in Section 2.9. Total RNA was extracted from SH-SY5Y cells treated with PrP^106-126^. The cDNAs were amplified using primers for the eight selected genes (Table S2).

### 2.16 Construction of protein-protein interaction (PPI) networks

The STRING online database (http://string-db.org) was utilized to annotate functional interactions among the DEGs. Interactions with a combined score >0.4 were included, and isolated nodes were excluded from the network. The PPI network was visualized in Cytoscape (version 3.10.2), with each node representing a gene's protein product. The top 10 hub genes were identified using the Molecular Complex Detection (MCODE) algorithm in Cytoscape.

### 2.17 Statistical analysis

All bioinformatics calculations and statistical analyses were conducted using R programming (version 4.3.1, https://www. r-project. org/). Data analysis was performed using limma for differential expression analysis of genes. Count data were modeled using a negative binomial distribution to account for biological variability and sequencing depth differences. Size factors were estimated to normalize the data, and gene-wise dispersion parameters were calculated to model the relationship between the mean and variance of counts. Differential expression was assessed using a generalized linear model and the Wald test. Adjusted p-values (Benjamini-Hochberg correction) were used to control the false discovery rate. Genes with an adjusted p-value <0.05 and an absolute log2 fold change >1 were considered statistically significant.

Data analysis was performed using GraphPad Prism software. Unpaired Student's t-test (for two groups) and one-way ANOVA (for multiple groups) were used, followed by Tukey's post-hoc test. Data were expressed as mean ± standard deviation (SD). *P* < 0.05 was considered statistically significant.

## 3 Results

### 3.1 Characterization of ferroptosis hallmarks in sCJD and PrP^106-126^-treated SH-SY5Y cells

**Fig. 1:**
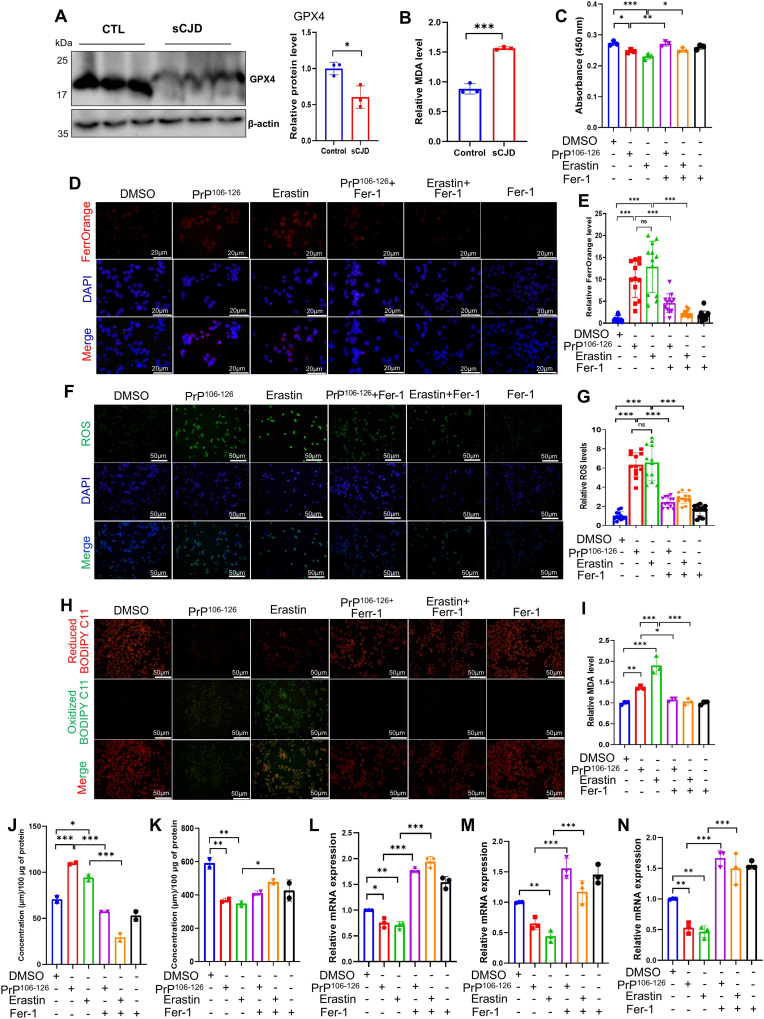
Altered expression of GPX4 and lipid peroxidation in sCJD patients with the induction of ferroptosis in SH-SY5Y cells by PrP^106-126^. (A) Representative Western blot showing GPX4 levels in the brain homogenates of sCJD patients and controls (n = 3). Quantification of the immunoblots in (A). (B) MDA content in the protein lysates from sCJD patients and controls. (C) Cell viability of SH-SY5Y cells treated with 100 μM PrP^106-126^ or 10 μM erastin and rescued with Fer-1 (2 μM) for 24 h, as measured by the CCK8 assay. (D) Iron accumulation in SH-SY5Y cells treated with PrP^106-126^. Fluorescence images of SH-SY5Y cells labeled with FerroOrange (red, Fe^2+^) and DAPI (blue, nuclei) after treatment with or without Fer-1 for 24 h. Scale bar = 20 μm. (E) Relative fluorescence intensity of FerroOrange. (F-G) Representative fluorescence microscopy image and quantitation of H_2_DCFDA-stained SH-SY5Y cells after 24 h of treatment. Green indicates H_2_DCFDA fluorescence and DAPI (blue, nuclei) (n = 3). Scale bar = 50 μm. (H) The effects of Fer-1 treatment on lipid peroxidation were evaluated by C11 BODIPY staining. SH-SY5Y cells were incubated with Fer-1 (2 μM) for 2 h, followed by treatment with PrP^106-126^ or erastin for 24 h. Scale bar = 50 μm. Images are representative of five independent experiments. (I) MDA content in treated SH-SY5Y and its reduction after Fer-1 treatment. (J) Levels of GSSG in treated SH-SY5Y cells. (K) Levels of reduced glutathione (GSH) in SH-SY5Y cells after 24 h of treatment. (L-N) mRNA expression levels of mGPX4, nGPX4 and SLC7A11 in treated SH-SY5Y cells. The data are presented as mean ± SD. Statistical significance was assessed using Student's two-tailed *t*-test or one-way ANOVA followed by Tukey's post-hoc multiple comparisons test. ∗*p* < 0.05; ∗∗*p* < 0.01; ∗∗∗*p* < 0.001.

In our previous study, we demonstrated that PrP expression levels were similar between sCJD patients and matched controls. However, PrP^Sc^ accumulation was observed in the sCJD patients but not in the matched controls [29]. Given the critical role of GPX4 in ferroptosis by suppressing lipid peroxidation, we assessed its status during sCJD progression. Western blot analysis revealed a significant decrease in GPX4 levels in sCJD brain tissue compared to controls (*p* < 0.05, Fig. 1A). The results showed that MDA levels were significantly higher in sCJD samples than in controls (*p* < 0.001; Fig. 1B), indicating that sCJD is associated with increased lipid peroxidation. This pattern of GPX4 depletion and increased lipid peroxidation supports the established hallmarks of ferroptosis, as seen in models such as erastin treatment.

Although changes in GPX4 and MDA levels in sCJD brain tissue suggest a ferroptosis-related environment, we used the PrP^106-126^ cellular model to examine whether these markers are associated with cell death-related processes. To assess its toxicity, SH-SY5Y cells were treated with PrP^106–126^, and cell viability was evaluated using CCK-8 and LDH assays. Consistent with previous findings, 24-h exposure to 100 μM PrP^106-126^ remarkably reduced SH-SY5Y cell viability [5,25]. Similarly, the CCK-8 assay indicated a significant decrease in SH-SY5Y cell viability after 24-h exposure to 100 μM PrP^106-126^ or 10 μM erastin, a ferroptosis inducer (*p* < 0.05, *p* < 0.001, respectively, Fig. 1C). To evaluate ferroptosis suppression by a lipophilic antioxidant, SH-SY5Y cells were pretreated with Fer-1 for 2 h before exposure to PrP^106-126^ or erastin. Pretreatment with Fer-1 significantly increased the viability of cells treated with PrP^106-126^ (*p* < 0.01) or erastin (*p* < 0.05) compared to cells not pretreated before application of PrP^106-126^ or erastin alone (Fig. 1C). These findings indicate that Fer-1 exerts a potential neuroprotective effect against PrP^106-126^-induced neurotoxicity in SH-SY5Y cells. No significant differences in cell viability were observed between the Fer-1-treated group and the negative control after 24 h (Fig. 1C). The CCK-8 assay results were further validated using the LDH release assay (Supplementary Fig. S1A). Bax is a cell death-promoting member of the Bcl-2 protein family. Consistently, we observed significantly elevated Bax protein expression in homogenized brain tissue from ME7-infected mice (Supplementary Fig. S1B and C).

As ferroptotic cell death involves cellular Fe^2+^ accumulation, we used a FerroOrange probe, which specifically reacts with Fe^2+^ in living cells, to monitor Fe^2+^ levels in treated SH-SY5Y cells (Fig. 1D). The findings indicated that PrP^106-126^ treatment significantly increased intracellular Fe^2+^ accumulation in SH-SY5Y cells (*p* < 0.001, Fig. 1D and E). Similarly, erastin treatment led to a significant increase in intracellular Fe^2+^ levels (*p* < 0.001, Fig. 1D and E). In contrast, Fer-1 significantly reduced cellular Fe^2+^ levels compared to the PrP^106-126^ or erastin groups (*p* < 0.001, Fig. 1D and E).

To further examine the potential involvement of ferroptosis in prion disease neurodegeneration, oxidative stress was assessed in SH-SY5Y cells. An H_2_DCFDA probe was used to assess intracellular ROS levels (Fig. 1F). Pretreatment with Fer-1 significantly decreased intracellular ROS accumulation compared to PrP^106-126^ or erastin treatment alone (*p* < 0.001, Fig. 1F and G). In addition, lipid peroxidation was detected by BODIPY 581/591C11, a fluorescent probe commonly used in ferroptosis studies. The results showed that PrP^106-126^ and erastin induced lipid peroxidation, which was significantly attenuated by Fer-1 treatment (Fig. 1H). Consistently, PrP^106-126^ and/or erastin also increased MDA content and glutathione disulfide (GSSG) levels (*p* < 0.01 and *p* < 0.001, respectively; Fig. 1I and J). Notably, PrP^106-126^ and/or erastin significantly decreased GSH levels (*p* < 0.01, Fig. 1K). Fer-1 pretreatment decreased MDA levels and increased GSH levels compared to PrP^106-126^ or erastin treatment alone (Fig. 1I–K). These findings confirm that Fer-1 plays a key role in protecting against ferroptosis induced by the neuropeptide PrP^106-126^.

Furthermore, mRNA levels of FRGs were assessed in treated SH-SY5Y cells. The protective effects of Fer-1 were evaluated by its ability to reverse PrP^106-126^- or erastin-induced changes in ferroptosis inhibitor mRNA levels (Fig. 1L–N). Fer-1 significantly restored the mRNA levels of mGPX4, nGPX4, and solute carrier family 7 member 11 (SLC7A11) (*p* < 0.001 for all, Fig. 1L–N), which were reduced by PrP^106-126^ or erastin. Expression of all GPX4 was significantly increased in the Fer-1-treated group compared to PrP^106-126^ or erastin treatment alone (*p* < 0.001, Supplementary Fig. S2). Taken together, these findings indicate that changes in ferroptosis markers observed after PrP^106-126^ treatment were consistent with the hallmarks of classic ferroptosis.

### 3.2 Ferroptosis-associated alterations in the brains of ME7-infected mice

The neurotropic ME7 strain is commonly used as a laboratory mouse scrapie model. To establish the prion disease model, ME7 scrapie from terminally ill mice was injected intraperitoneally into healthy mice. Our previous study showed that the body weight of ME7-infected mice significantly decreased, and these mice displayed progressive neurological symptoms, leading to death at 7 months post-injection [30]. The conversion of PrP^C^ to PrP^Sc^ is considered responsible for prion disease. Thus, we examined PrP^Sc^ levels by Western blot analysis. The findings demonstrated that ME7-infected mice exhibited a high accumulation of PrP^Sc^, whereas no detectable levels of PrP^Sc^ were observed in the control group (Supplementary Fig. S3A). In addition, the abnormal behaviors and elevated PrP^Sc^ in ME7-infected mice were accompanied by increased expression of GFAP, as shown by Western blot analysis and immunofluorescence staining (Supplementary Fig. S3 B, C) [25,31].

**Fig. 2:**
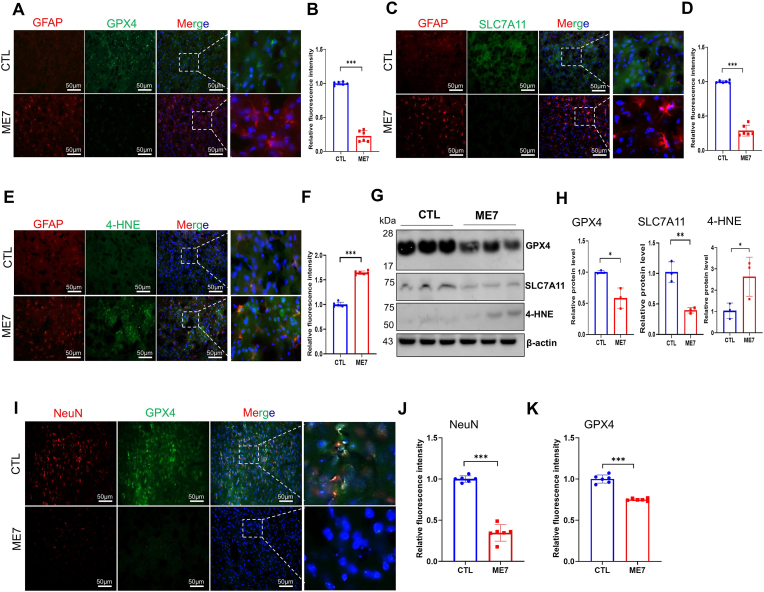
GPX4 and SLC7A11 expressions decreased in impaired astrocytes of ME7-infected mice, meanwhile, 4-HNE increased. (A-B) Representative immunofluorescence image and relative fluorescence intensity of GPX4 expression in the thalamus region of ME7-infected mice or non-infected controls (CTL), showing GPX4 (green) in astrocytes expressing the marker GFAP (red) (n = 3 mice per group). Nuclei stained with DAPI are shown in blue. Scale bar = 50 μm. (C-D) Representative immunofluorescence images and relative fluorescence intensity of SLC7A11 expression in the thalamus region of brains from ME7-infected mice or non-infected controls (CTL), showing SLC7A11 (green) in astrocytes expressing the marker GFAP (red) (n = 3 mice per group). Nuclei stained with DAPI are shown in blue. Scale bar = 50 μm. (E-F) Representative immunofluorescence images and relative fluorescence intensity of 4-HNE protein expression in the thalamus region of ME7-infected mice or non-infected controls (CTL), showing 4-HNE (green) in astrocytes expressing the marker GFAP (red) (n = 3 mice per group). Nuclei stained with DAPI are shown in blue. Scale bar = 50 μm. (G) Representative western blots showing GPX4, SLC7A11, and 4-HNE levels in the brain homogenates of ME7-infected and control mice. (H) Quantification of the immunoblots in (G). (I) Immunofluorescence image showing GPX4 (green) and NeuN (red) expression in the thalamus region of ME7-infected mice or non-infected controls (CTL). Scale bar = 50 μm. (J-K) Relative fluorescence intensity of NeuN and GPX4 compared to controls. Data are presented as the mean ± SD. ∗*p* < 0.05, ∗∗*p* < 0.01, ∗∗∗*p* < 0.001 by Student's two-tailed t-test GPX4: Glutathione peroxidase 4, SLC7A11: Solute carrier family 7 member 11, 4-HNE: 4-Hydroxynonenal, NeuN: Neuronal nuclei, GPX4: Glutathione peroxidase 4.

To further explore whether ferroptosis contributes to ME7-induced prion disease, we examined ferroptosis-related alterations in the thalamus of ME7-infected mice compared with non-infected control mice. The SLC7A11/GPX4 pathway serves as a defense mechanism against ferroptosis by promoting intracellular GSH synthesis and mitigating lipid peroxidation. Thus, we investigated its potential role in prion disease progression using immunofluorescence staining of whole-brain sections from ME7-infected mice. Given that reactive astrocytosis is a well-established hallmark of prion disease, we co-stained sections with GFAP alongside ferroptosis-related markers to determine whether thalamic regions exhibiting astrocytosis simultaneously display a ferroptosis signature. The results demonstrated a significant reduction in GPX4 and SLC7A11 immunofluorescence intensity in thalamic regions of ME7-infected mice, where prominent GFAP-positive reactive astrocytosis was simultaneously observed, compared to controls (*p* < 0.001) (Fig. 2A–D). These findings were corroborated by Western blot analysis, which showed significant reductions in GPX4 (*p* < 0.05) and SLC7A11 (*p* < 0.01) protein levels in ME7-infected mice (Fig. 2G and H).

We then investigated whether oxidative stress-related lipid peroxidation levels were elevated in thalamic regions of ME7-infected mice exhibiting reactive astrocytosis. Consequently, we assessed 4-HNE levels, a marker of oxidative stress-related lipid peroxidation and cytotoxicity, in whole-brain tissue sections from ME7-infected and non-infected control mice by immunofluorescence co-staining with GFAP. The immunofluorescence analysis revealed a significant increase in 4-HNE staining intensity in thalamic regions characterized by GFAP-positive reactive astrocytosis (*p < *0.001, Fig. 2E and F). The immunofluorescence result of 4-HNE was confirmed by Western blot analysis (Fig. 2G and H). Collectively, these results suggest that ferroptosis-related processes are involved in the brains of mice with prion disease, characterized by reduced GPX4 and SLC7A11 expression and increased 4-HNE levels. Therefore, these findings suggest that ferroptosis-associated alterations may contribute to neuronal damage during neurodegeneration in prion disease.

Neuronal damage is the primary risk factor contributing to the neurological sequelae observed in prion disease neurodegeneration. Previous studies have indicated that GPX4 plays a key role in redox balance by reducing lipid hydroperoxides [32]. Thus, GPX4 depletion may contribute to neuronal damage. As expected, GPX4 expression in neurons showed diminished neuronal nuclei (NeuN) and GPX4 staining in ME7-infected mice compared to non-infected control mice (Fig. 2I). Quantification of fluorescence density confirmed a significant reduction in NeuN and GPX4 levels in ME7-infected mice (*p* < 0.001, *p* < 0.001, respectively) (Fig. 2J and K), indicating severe neuronal damage in these mice. Together with simultaneous reductions in GPX4 and SLC7A11, increased 4-HNE levels, and functional rescue by Fer-1 in our *in vitro* model, these findings collectively suggest that ferroptosis-associated alterations contribute to neuronal damage in prion disease pathogenesis.

### 3.3 Transcriptomic analysis of sCJD patients reveals dysregulation of ferroptosis pathways

**Fig. 3:**
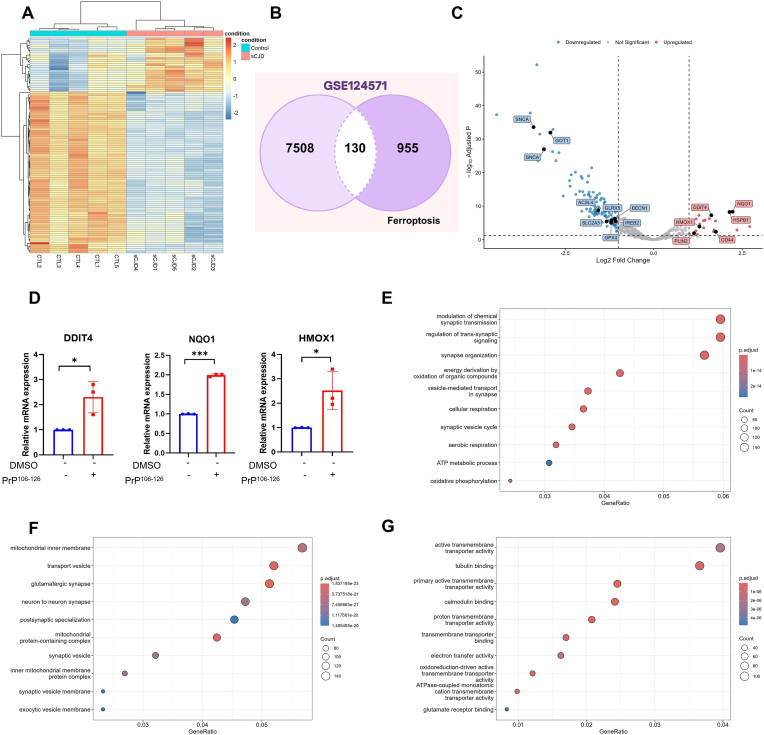
Identification of ferroptosis-related genes (FRGs). (A) Heatmap of the top 100 FRGs between sporadic Creutzfeldt-Jakob disease (sCJD) patients and controls (sCJD vs. control, CTL). (B) Venn diagram showing the intersection of FRGs from the GSE124571 dataset with the FerrDB database. (C) Volcano plot of differentially expressed FRGs from GSE124571 data analysis between sCJD and CTL. Upregulated genes are shown in red, and downregulated genes are shown in blue. (D) Relative expression levels of three randomly selected upregulated DEGs (NQO1, DDIPT4, and HMOX1) were significantly higher in treated SH-SY5Y cells than in non-treated cells. Data are presented as the mean ± SD. ∗*p* < 0.05, and ∗∗∗*p* < 0.001 by Student's two-tailed *t*-test. (E) Gene Ontology (GO) enrichment analysis of biological processes, (F) GO enrichment analysis of cell components, and (G) GO enrichment analysis of molecular function.

The gene expression matrix was obtained after data correction and standardization using limma. A total of 7638 DEGs were identified in sCJD patients compared to controls. To illustrate transcriptomic differences between the two groups, the expression levels of the top 100 upregulated and downregulated FRGs were visualized in a heatmap (Fig. 3A). To characterize the ferroptotic landscape, we intersected these DEGs with 1085 genes from the FerrDb database, identifying 130 significant FRGs, of which 21 were upregulated, and 109 were downregulated (Fig. 3B; Supplementary data 2). Volcano plot analysis further highlighted a significant downregulation of core ferroptosis-related regulators in sCJD, including GPX4, ACSL4, SNCA, GOT1, GLRX5, BECN1, IREB2, and SLC2A3 (Fig. 3C). This transcriptional profile supports a broad disturbance of lipid and iron homeostasis in sCJD. Notably, GPX4 was consistently downregulated across all three experimental models, while other ferroptosis-related regulators, including ACSL4, IREB2, and BECN1, showed significant dysregulation in the human transcriptomic dataset (Supplementary data 2). Furthermore, the upregulation of NQO1, HSPB1, DDIT4, PDK4, and HMOX1 underscores the activation of a robust ferroptotic program in sCJD patients [33]. Moreover, the expression levels of DDIT4, NQO1, and HMOX1 were significantly higher in treated SH-SY5Y than in non-treated cells, which was consistent with the transcriptomic data obtained by RNA-seq (Fig. 3D). The GO and KEGG pathway enrichment analyses were performed to investigate the biological mechanisms of the 130 FRGs. The analysis revealed significant enrichment of biological processes, including modulation of chemical synaptic transmission, regulation of trans-synaptic signaling and synapse organization, and energy derivation by oxidation processes (Fig. 3E). Cellular components were enriched in the mitochondrial inner membrane, transport vesicles, and mitochondrial protein-containing complexes (Fig. 3F). Molecular functions were enriched in active transmembrane transporter activity and tubulin binding (Fig. 3G).

**Fig. 4:**
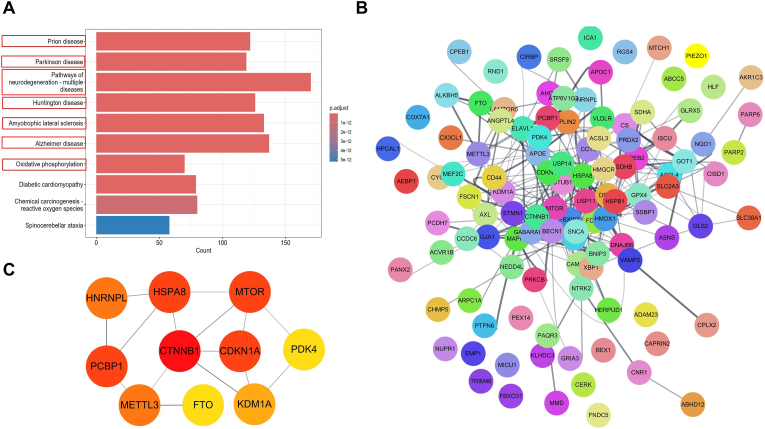
Pathway enrichment of identified ferroptosis-related genes (FRGs). (A) KEGG enrichment analysis of downregulated and upregulated FRGs (top 10 pathways). (B) Network of enriched terms generated using Metascape software. (C) Network diagram showing the top biological pathways identified by Metascape software.

The KEGG pathway enrichment analyses identified signaling pathways associated with neurodegenerative diseases, such as Parkinson's disease, Alzheimer's disease, and amyotrophic lateral sclerosis, as well as oxidative phosphorylation (Fig. 4A). To further explore interactions among DEGs, 130 genes were uploaded to the STRING database, and a PPI network was constructed using Cytoscape (Fig. 4B). In addition, the CytoHubba plugin was used to identify key modules. The top 10 hub genes were identified: PDK4, MTOR, HSPA8, PCBP1, METTL3, FTO, CTNNB1, KDM1A, HNRNPL, and CDKN1A (Fig. 4C). Several of these hub genes have been reported to function as upstream regulators within ferroptosis-associated pathways that may influence GPX4 activity or ferroptosis susceptibility (Supplementary Fig. S4).

## 4 Discussion

Although prion diseases are uncommon, they are typically lethal and lack effective treatments. Ferroptosis is increasingly recognized as a contributing factor in neurodegenerative diseases. Identifying the specific factors that drive ferroptosis in prion diseases could provide novel therapeutic targets and strategies to address these lethal neurodegenerative disorders. In this study, we investigated the role of ferroptosis in the progression of sCJD and in the ME7 scrapie strain mouse model. Our biochemical analyses revealed a strong association between ferroptosis and prion diseases. Specifically, SH-SY5Y cells exposed to PrP^106-126^ exhibited elevated levels of intracellular Fe^2+^, ROS, GSSG, and lipid peroxidation, accompanied by decreased GSH levels. Pretreatment with Fer-1 effectively protected SH-SY5Y cells from ferroptosis induced by either PrP^106-126^ or erastin. Furthermore, Western blot analysis showed that GPX4 and SLC7A11 expression levels were significantly reduced in ME7-infected mice compared to non-infected controls. Immunofluorescence co-staining confirmed that these reductions were evident in thalamic regions exhibiting prominent GFAP-positive reactive astrocytosis. In contrast, levels of 4-HNE, a marker of lipid peroxidation and an inducer of ferroptosis, were markedly increased. Transcriptomic analysis of the GSE124571 dataset identified 130 notable FRGs, comprising 21 upregulated and 109 downregulated genes. These findings further support a correlation between ferroptosis and the progression of prion diseases.

GPX4 is a crucial enzyme in regulating ferroptosis [32,34], serving as a master regulator in this process [11]. Therefore, the role of GPX4 is particularly relevant in the context of ferroptosis in prion diseases. First, we showed that GPX4 expression decreased in the sCJD brain compared with the control. Additionally, MDA levels increased in sCJD samples, ultimately leading to cell death. MDA is a secondary byproduct of ferroptosis [14]. The susceptibility of neurons in ME7-infected mice to ferroptosis was confirmed by decreased GPX4 and SLC7A11 expression in the thalamus region of ME7-infected mice. SLC7A11/GPX4 pathway functions act as a defense mechanism against ferroptosis [35]. Therefore, we speculate that decreased GPX4 and SLC7A11 expression may compromise the antioxidant defense within the prion-affected brain microenvironment, contributing to ferroptosis-associated neuronal damage during disease progression. Consistent with this, immunofluorescence analysis revealed a significant reduction in NeuN-positive neurons, accompanied by simultaneous downregulation of GPX4, in thalamic regions of ME7-infected mice. These findings are consistent with neurodegenerative diseases in which low GPX4 expression in neurons can lead to ferroptotic damage, such as in Alzheimer's disease and experimental cerebral malaria models [36].

Lipid peroxidation is a hallmark of ferroptosis. Pathological events in neurodegenerative diseases, such as lipid peroxidation, are important features of ferroptosis. Due to the high levels of PUFAs in the brain, lipid peroxidation is hypothesized to be a significant occurrence of ferroptosis in prion diseases. This hypothesis is based on elevated lipid peroxidation levels in cerebrospinal fluid and plasma in patients with sCJD [37]. Bate et al. reported that treatment of neuronal cell lines with PUFAs, particularly susceptible to peroxidation, significantly increased PrP^Sc^ formation [38]. This high level of lipid peroxidation, as the driving force of ferroptosis, can disrupt cellular function. Thus, it is essential to investigate 4-HNE, a chemically reactive aldehyde and an abundant, diffusible product of lipid peroxidation, which exerts various cytotoxic effects, including GSH depletion and induction of cell death [39]. In prion diseases, such as human sCJD patients and scrapie-infected mice, 4-HNE is produced in the brains [40]. Astrocytes, rather than neurons, have been found to contain 4-HNE adducts, and their role in the progression of prion disease remains unclear [41]. Our findings corroborate earlier research demonstrating that elevated levels of 4-HNE are associated with neuropathological changes in prion-affected brain regions. Additionally, these results are consistent with the reported increase in lipid peroxidation in SH-SY5Y cells treated with PrP^106-126^.

Studies show that PrP^C^ itself may influence iron metabolism and has been implicated in the regulation of iron uptake and storage [42]. Moreover, iron sequestration in PrP^Sc^ aggregates leads to a phenotype of iron deficiency and is thought to be the primary reason for iron metabolism dysregulation in diseased brains [43]. The presence of iron-induced oxidative stress in mouse models has been linked to the neurodegeneration in prion disease [44]. Thus, changes in iron metabolism are also believed to play a role in the development of neurodegeneration and the progression of prion diseases. Therefore, certain protective mechanisms may arise against iron-induced oxidative damage [21]. Our results are consistent with previous studies [14,35], detected intracellular Fe^2+^, ROS production, and lipid peroxidation-hallmark signs of ferroptosis-in SH-SY5Y cells treated with PrP^106-126^. Fer-1, an aromatic amine that specifically binds to lipid ROS, protects cells from lipid peroxidation [45]. Fer-1 inhibited PrP^106-126^-induced neurotoxicity and cell death, suggesting a link between PrP^106-126^-induced cell death and ferroptosis. We also confirmed that Fer-1 effectively blocked erastin-triggered ferroptosis. Consequently, PrP^106-126^-induced ferroptotic cell death exhibited characteristics similar to those of ferroptosis, including susceptibility to lipid peroxidation and inhibition by Fer-1.

In the meantime, analysis of the GEO database of brain tissue from sCJD patients showed that ferroptosis-suppressor genes were downregulated, while ferroptosis-driver genes were upregulated. The results of the GO functional enrichment analysis revealed enrichment in biological processes, including modulation of chemical synaptic transmission, mitochondrial inner membrane function, glutamatergic synapse activity, neuron-to-neuron synapse formation, and active transmembrane transporter activity pathways. Further analysis using KEGG pathway enrichment revealed that the differentially expressed FRGs were predominantly enriched in pathways related to neurodegenerative diseases. To demonstrate how the identified hub genes are functionally related to GPX4, we created a network diagram showing that the top 10 hub genes act as upstream regulatory nodes, covering metabolic, epigenetic, iron homeostasis, and signalling pathways, which all integrate on GPX4 as the final effector of ferroptosis [[46], [47], [48], [49], [50], [51], [52]]. The hub gene analysis in this study is exploratory. Future multicenter studies with larger sCJD cohorts are necessary to better define the upstream regulatory network of ferroptosis in prion diseases.

While the undifferentiated SH-SY5Y neuroblastoma model was used to demonstrate PrP^106-126^-induced ferroptosis, it is important to acknowledge the limitations of this cell line, including its reported resistance to specific inducers, such as erastin [53], and its immaturity compared to primary neurons. Future studies should confirm these key mechanisms in more physiologically relevant models, such as differentiated neuronal cultures. A key limitation of the current study is that neuroprotection was demonstrated *in vitro* using PrP^106-126^-induced toxicity; the *in vivo* efficacy of Fer-1 in prion-infected animal models or conditional neuronal GPX4 knockout models has not been thoroughly assessed. In conclusion, our biochemical analyses suggest a strong correlation, rather than direct causality, between ferroptosis and prion diseases. These observations imply that ferroptotic injury to neurons may contribute to neurological issues in survivors of prion diseases, opening avenues for future exploration of ferroptosis-modulating strategies as potential adjuvant approaches.

## Funding

This work was supported by the Basic Science Research Program through the 10.13039/501100001321National Research Foundation (NRF) of Korea, which is funded by the 10.13039/100009950Ministry of Education (2017R1A6A1A03015876, RS-2025-00517133, RS-2025-24792972, RS-2025-23963916). It was also supported by a grant from the 10.13039/501100003716Korea Basic Science Institute (National Research Facilities and Equipment Center), funded by the 10.13039/100009950Ministry of Education (grant no. RS-2021-NF000550).

## CRediT authorship contribution statement

**Mohammed Zayed:** Conceptualization, Data curation, Formal analysis, Methodology, Writing – original draft. **Hilal Tayara:** Formal analysis, Investigation, Methodology, Software. **Byung-Hoon Jeong:** Conceptualization, Formal analysis, Funding acquisition, Methodology, Project administration, Validation, Writing – review & editing.

## Declaration of competing interest

The authors declare that they have no known competing financial interests or personal relationships that could have appeared to influence the work reported in this paper.

## Data Availability

All data are reported in the article and supplementary materials.

## References

[1] Scheckel C., Aguzzi A. (2018). Prions, prionoids and protein misfolding disorders. Nat. Rev. Genet..

[2] Appleby B.S., Connor A., Wang H. (2019). Therapeutic strategies for prion disease: a practical perspective. Curr. Opin. Pharmacol..

[3] Zayed M., Kook S.H., Jeong B.H. (2023). Potential therapeutic use of stem cells for prion diseases. Cells.

[4] Chiesa R. (2015). The elusive role of the prion protein and the mechanism of toxicity in prion disease. PLoS Pathog..

[5] Zayed M., Jeong B.H. (2024). Adipose-derived mesenchymal stem cell secretome attenuates prion protein peptide (106-126)-Induced oxidative stress via Nrf2 activation. Stem Cell Rev. Rep..

[6] Tahir W., Zafar S., Llorens F. (2018). Molecular alterations in the cerebellum of sporadic Creutzfeldt-Jakob disease subtypes with DJ-1 as a key regulator of oxidative stress. Mol. Neurobiol..

[7] Shah S.Z.A., Zhao D., Hussain T. (2018). p62-Keap1-NRF2-ARE pathway: a contentious player for selective targeting of autophagy, oxidative stress and mitochondrial dysfunction in prion diseases. Front. Mol. Neurosci..

[8] Brazier M.W., Lewis V., Ciccotosto G.D. (2006). Correlative studies support lipid peroxidation is linked to PrP(res) propagation as an early primary pathogenic event in prion disease. Brain Res. Bull..

[9] López-Pérez Ó., Badiola J.J., Bolea R. (2020). An update on autophagy in prion diseases. Front. Bioeng. Biotechnol..

[10] Zafar S., Behrens C., Dihazi H. (2018). Cellular prion protein mediates early apoptotic proteome alternation and phospho-modification in human neuroblastoma cells. Cell Death Dis..

[11] Dixon S.J., Lemberg K.M., Lamprecht M.R. (2012). Ferroptosis: an iron-dependent form of nonapoptotic cell death. Cell.

[12] Cerasuolo M., Di Meo I., Auriemma M.C. (2023). Iron and ferroptosis more than a suspect: beyond the Most common mechanisms of neurodegeneration for new therapeutic approaches to cognitive decline and dementia. Int. J. Mol. Sci..

[13] Wang Y., Wu S., Li Q. (2023). Pharmacological inhibition of ferroptosis as a therapeutic target for neurodegenerative diseases and strokes. Adv. Sci. (Weinh.).

[14] Yang W.S., Stockwell B.R. (2016). Ferroptosis: death by lipid peroxidation. Trends Cell Biol..

[15] Li J., Cao F., Yin H-l (2020). Ferroptosis: past, present and future. Cell Death Dis..

[16] Su L.J., Zhang J.H., Gomez H. (2019). Reactive oxygen species-induced lipid peroxidation in apoptosis, autophagy, and ferroptosis. Oxid. Med. Cell. Longev..

[17] Thirupathi A., Chang Y.Z. (2019). Brain iron metabolism and CNS diseases. Adv. Exp. Med. Biol..

[18] Chaudhary S., Ashok A., Wise A.S. (2021). Upregulation of brain hepcidin in prion diseases. Prion.

[19] Singh A., Qing L., Kong Q. (2012). Change in the characteristics of ferritin induces iron imbalance in prion disease affected brains. Neurobiol. Dis..

[20] Singh A., Isaac A.O., Luo X. (2009). Abnormal brain iron homeostasis in human and animal prion disorders. PLoS Pathog..

[21] Kim B.H., Jun Y.C., Jin J.K. (2007). Alteration of iron regulatory proteins (IRP1 and IRP2) and ferritin in the brains of scrapie-infected mice. Neurosci. Lett..

[22] Singh N. (2014). The role of iron in prion disease and other neurodegenerative diseases. PLoS Pathog..

[23] Lin C.F., Yu K.H., Jheng C.P. (2013). Curcumin reduces amyloid fibrillation of prion protein and decreases reactive oxidative stress. Pathogens.

[24] Ou M., Jiang Y., Ji Y. (2022). Role and mechanism of ferroptosis in neurological diseases. Mol. Metabol..

[25] Zayed M., Kim Y.C., Jeong B.H. (2024). Assessment of the therapeutic potential of Hsp70 activator against prion diseases using in vitro and in vivo models. Front. Cell Dev. Biol..

[26] Ito K., Eguchi Y., Imagawa Y. (2017). MPP+ induces necrostatin-1- and ferrostatin-1-sensitive necrotic death of neuronal SH-SY5Y cells. Cell Death Discov..

[27] Zhang Y., Fan B.Y., Pang Y.L. (2020). Neuroprotective effect of deferoxamine on erastininduced ferroptosis in primary cortical neurons. Neural Regen. Res..

[28] Wang Y.-Q., Chang S.-Y., Wu Q. (2016). The Protective Role of Mitochondrial Ferritin on Erastin-Induced Ferroptosis [Original Research]. Front. Aging Neurosci..

[29] Kim Y.C., Won S.Y., Jeong B.H. (2021). Altered expression of glymphatic system-related proteins in prion diseases: implications for the role of the glymphatic system in prion diseases. Cell. Mol. Immunol..

[30] Sim H.-J., Kim Y.-C., Bhattarai G. (2023). Prion infection modulates hematopoietic stem/progenitor cell fate through cell-autonomous and non-autonomous mechanisms. Leukemia.

[31] Zayed M., Kim Y.-C., Jeong B.-H. (2025). Therapeutic effects of adipose-derived mesenchymal stem cells combined with glymphatic system activation in prion disease. Mol. Neurodegener..

[32] Xu C., Sun S., Johnson T. (2021). The glutathione peroxidase Gpx4 prevents lipid peroxidation and ferroptosis to sustain Treg cell activation and suppression of antitumor immunity. Cell Rep..

[33] Fei Y., Ding Y. (2024). The role of ferroptosis in neurodegenerative diseases. Front. Cell. Neurosci..

[34] Imai H., Nakagawa Y. (2003). Biological significance of phospholipid hydroperoxide glutathione peroxidase (PHGPx, GPx4) in mammalian cells. Free Radic. Biol. Med..

[35] Tang D., Chen X., Kang R. (2021). Ferroptosis: molecular mechanisms and health implications. Cell Res..

[36] Yang S., Xie Z., Pei T. (2022). Salidroside attenuates neuronal ferroptosis by activating the Nrf2/HO1 signaling pathway in Aβ(1-42)-induced Alzheimer's disease mice and glutamate-injured HT22 cells. Chin. Med..

[37] Arlt S., Kontush A., Zerr I. (2002). Increased lipid peroxidation in cerebrospinal fluid and plasma from patients with Creutzfeldt-Jakob disease. Neurobiol. Dis..

[38] Bate C., Tayebi M., Diomede L. (2008). Docosahexaenoic and eicosapentaenoic acids increase prion formation in neuronal cells. BMC Biol..

[39] Ayala A., Muñoz M.F., Argüelles S. (2014). Lipid peroxidation: production, metabolism, and signaling mechanisms of malondialdehyde and 4-hydroxy-2-nonenal. Oxid. Med. Cell. Longev..

[40] Cassard H., Huor A., Espinosa J.C. (2020). Prions from sporadic Creutzfeldt-Jakob disease patients propagate as strain mixtures. mBio.

[41] Andreoletti O., Levavasseur E., Uro-Coste E. (2002). Astrocytes accumulate 4-hydroxynonenal adducts in murine scrapie and human Creutzfeldt-Jakob disease. Neurobiol. Dis..

[42] Singh A., Mohan M.L., Isaac A.O. (2009). Prion protein modulates cellular iron uptake: a novel function with implications for prion disease pathogenesis. PLoS One.

[43] Singh N., Haldar S., Tripathi A.K. (2014). Iron in neurodegenerative disorders of protein misfolding: a case of prion disorders and Parkinson's disease. Antioxidants Redox Signal..

[44] Kim N.H., Park S.J., Jin J.K. (2000). Increased ferric iron content and iron-induced oxidative stress in the brains of scrapie-infected mice. Brain Res..

[45] Cao J.Y., Dixon S.J. (2016). Mechanisms of ferroptosis. Cell. Mol. Life Sci..

[46] Song X., Liu J., Kuang F. (2021). PDK4 dictates metabolic resistance to ferroptosis by suppressing pyruvate oxidation and fatty acid synthesis. Cell Rep..

[47] Zhang Y., Swanda R.V., Nie L. (2021). mTORC1 couples cyst(e)ine availability with GPX4 protein synthesis and ferroptosis regulation. Nat. Commun..

[48] Zhou J., Tan Y., Hu L. (2022). Inhibition of HSPA8 by rifampicin contributes to ferroptosis via enhancing autophagy. Liver Int..

[49] Yang Z., Yan Y., Wang L. (2025). PCBP1 modulates cellular iron homeostasis via targeting HIF-1α/HO-1 pathway and alleviates high-glucose-induced ferroptosis in HRMECs. Exp. Eye Res..

[50] Tang Y., Li Z., Zhao X. (2025). METTL3-mediated m6A modification promotes ferroptosis in adenomyosis through GPX4 in a YTHDF1-dependent manner. Reproduction.

[51] Wang H., Zhang H., Chen Y. (2022). Targeting Wnt/β-Catenin signaling exacerbates ferroptosis and increases the efficacy of melanoma immunotherapy via the regulation of MITF. Cells.

[52] Tarangelo A., Dixon S. (2018). The p53-p21 pathway inhibits ferroptosis during metabolic stress. Oncotarget.

[53] Cardona C.J., Kim Y., Chowanadisai W. (2024). Considerations for using neuroblastoma cell lines to examine the roles of iron and ferroptosis in neurodegeneration. Cells.

