# Supplementary material for: Ferroptosis-related mechanisms in prion diseases provide insights into neurodegeneration and reveal therapeutic implications
Source: Redox Biol. 2026 Apr 4;93:104155. doi: 10.1016/j.redox.2026.104155 (PMC13090729; doi:10.1016/j.redox.2026.104155)
Supplement: Multimedia component 1 [file mmc1.docx]

**Ferroptosis-related mechanisms in prion diseases: Insights into neurodegeneration and therapeutic implications**

**Mohammed Zayed^a, b, c^, Hilal Tayara^d^, Byung-Hoon Jeong^a, b*^**

^a^ Korea Zoonosis Research Institute, Jeonbuk National University, Iksan 54531, Republic of Korea

^b^ Department of Bioactive Material Sciences, Jeonbuk National University, Jeonju 54896, Republic of Korea

^c^ Department of Surgery, College of Veterinary Medicine, Qena University, Qena 83523, Egypt

^d^ School of International Engineering and Science, Jeonbuk National University, Jeonju, 54896, Republic of Korea.

***** Corresponding author

Korea Zoonosis Research Institute, Jeonbuk National University, 820-120, Hana-ro, Iksan 54531, Republic of Korea

E-mail address: [bhjeong@jbnu.ac.kr](mailto:bhjeong@jbnu.ac.kr) (Byung-Hoon Jeong)


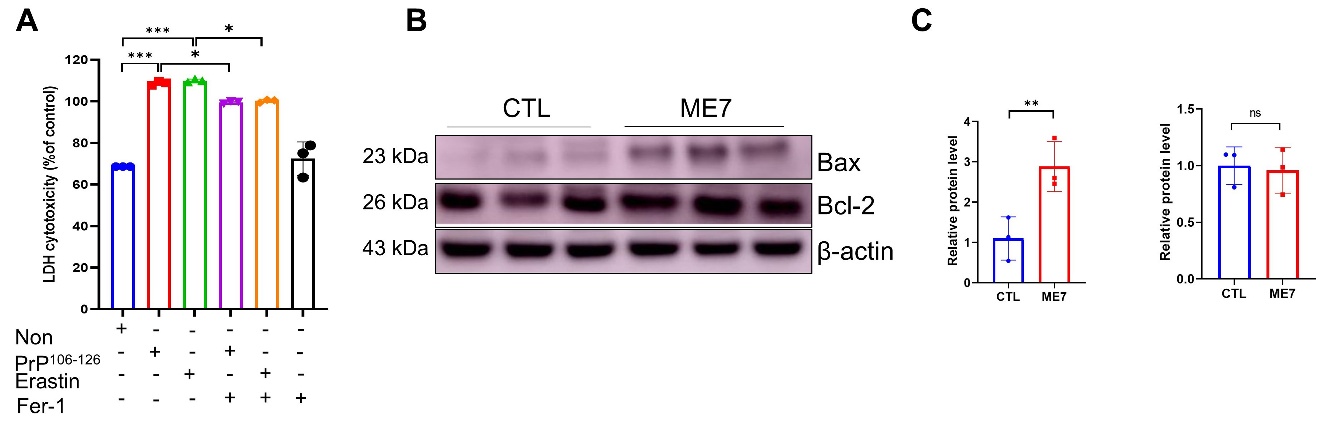
**Figure S1:** (A) Cytotoxicity assay of SH-SY5Y cells treated with 10 μM PrP^106-126^ or 10 μM erastin and rescued with fer-1 (2 μM) for 24 hours, measured by the lactate dehydrogenase (LDH) assay. Data are expressed as mean ± standard deviation. *** *p*<0.001. A one-way ANOVA was used to compare multiple groups. (B-C) Western blotting for determination of Bax and Bcl-2 in brain homogenates from ME7-infected mice compared to control non-infected (CTL). Data are presented as the mean ± standard deviation (n = 3). ***p* < 0.01 by Student’s two-tailed t-test.


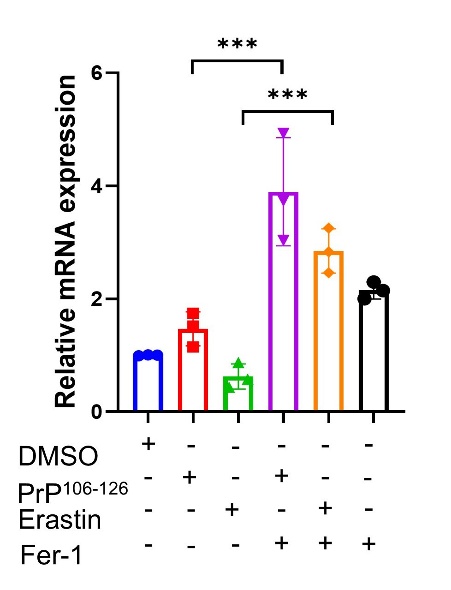


**Figure S2:** mRNA expression levels of allGPX4 in treated SH-SY5Y cells. Data are presented as mean ± standard deviation (n = 3). Statistical significance was tested using one-way ANOVA with post-hoc Tukey’s multiple comparisons test. ****p* < 0.001.


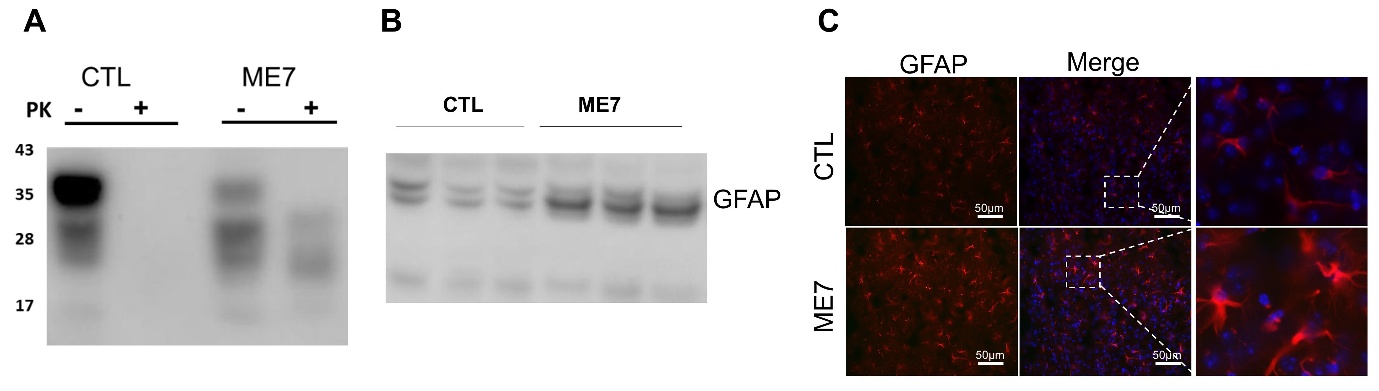


**Figure S3:** (A) Western blot analysis to detect both total PrP and the disease-associated proteinase-K (PK) resistant infectious PrP (PrP^Sc^) between ME7-infected mice (ME7) and non-infected control mice (CTL). (B) Representative immunoblot of GFAP. (C) Representative immunofluorescence images of GFAP expression in the thalamus region from ME7-infected mice or non-infected controls (CTL). GFAP (red) (n = 3 per group). Nuclei stained with DAPI are shown in blue. Scale bar = 50 μm.


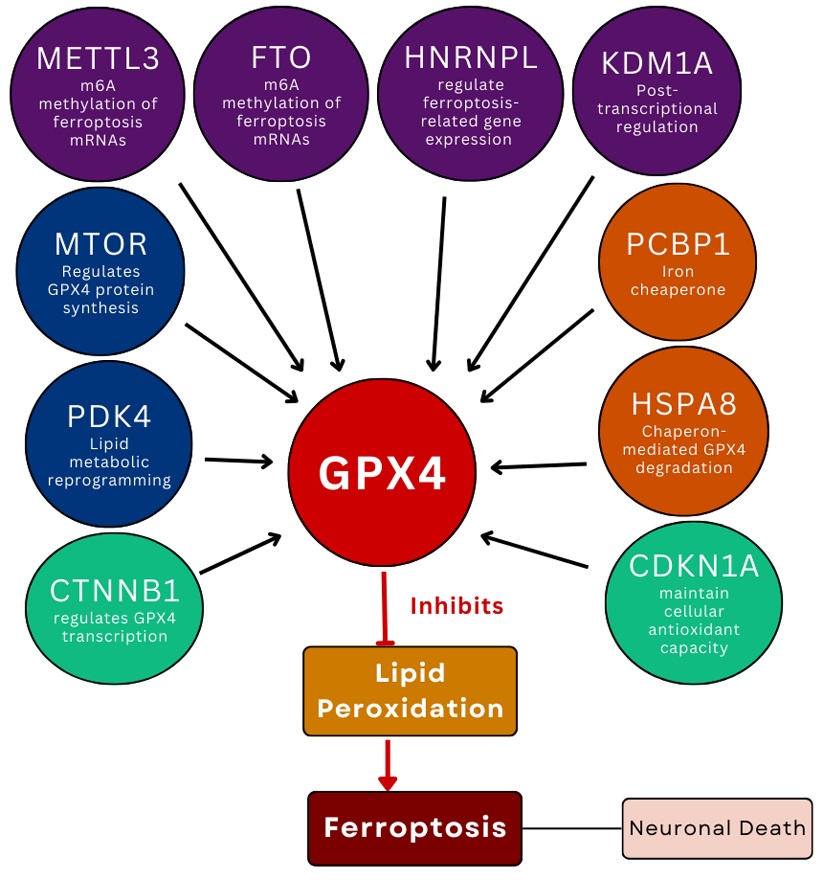


**Figure S4: Regulatory network illustrating potential upstream relationships between the identified hub genes and GPX4 within ferroptosis-associated pathways.**

Hub genes were identified by CytoHubba analysis from the PPI network of 130 ferroptosis-related DEGs in sCJD patients (GSE124571).

**Table S1:** Detailed information of the sporadic Creutzfeldt-Jakob disease (CJD) patients and age and sex-matched controls investigated in this study.

|  | Characteristics | Age | Sex | *PRNP* codon 129 |
| --- | --- | --- | --- | --- |
| CJD1 | Sporadic CJD | 60 | Female | MM |
| CJD2 | Sporadic CJD | 72 | Female | MM |
| CJD3 | Sporadic CJD | 86 | Male | MM |
|  |  |  |  |  |
| CTL1 | Controls | 57 | Female | MM |
| CTL2 | Controls | 73 | Female | MM |
| CTL3 | Controls | 84 | Male | VV |

**Table S2** The primer sequences used for real-time PCR.

| **Gene** | **Forward primer** | **Reverse primer** |
| --- | --- | --- |
| allGPX4 | TGTGCGCGCTCCATGCACGACT | CGAATTTGACGTTGTAGCCCG |
| mGPX4 | CTCGGCCGCCTTTGCCGCCTA | CGAATTTGACGTTGTAGCCCG |
| nGPX4 | CCGGCGGAAGAAGCCCTGTCC | CGAATTTGACGTTGTAGCCCG |
| SLC7A11 | CGGGGCGTATTACCAGCAGT | CAATGGACGCTTCTTGGATGG |
| NQO1  HMOX1  DDIT4 | CCCTGCGAACTTTCAGTATCC  GGAACTTTCAGAAGGGCCAG  CGGAGGAAGACACGGCTTACC | CTTTCAGAATGGCAGGGACTC  GGAAGTAGACAGGGGCGAAG  GCTTACCAACTGGCTAGGCATCA |
