# Supplementary figures and images for: Ferroptosis-related mechanisms in prion diseases provide insights into neurodegeneration and reveal therapeutic implications
Source: Redox Biol. 2026 Apr 4;93:104155. doi: 10.1016/j.redox.2026.104155 (PMC13090729; doi:10.1016/j.redox.2026.104155)

Uncropped Western blots for Fig. 1 A

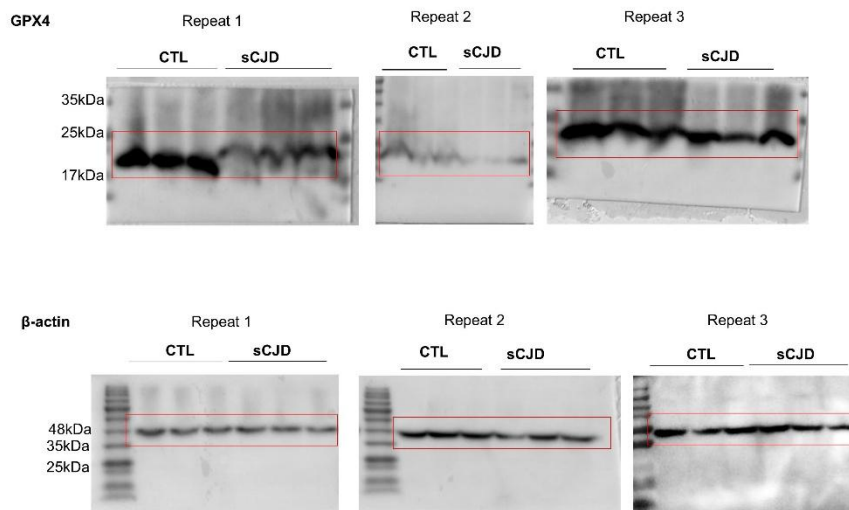

Uncropped Western blots for Fig. 2 G

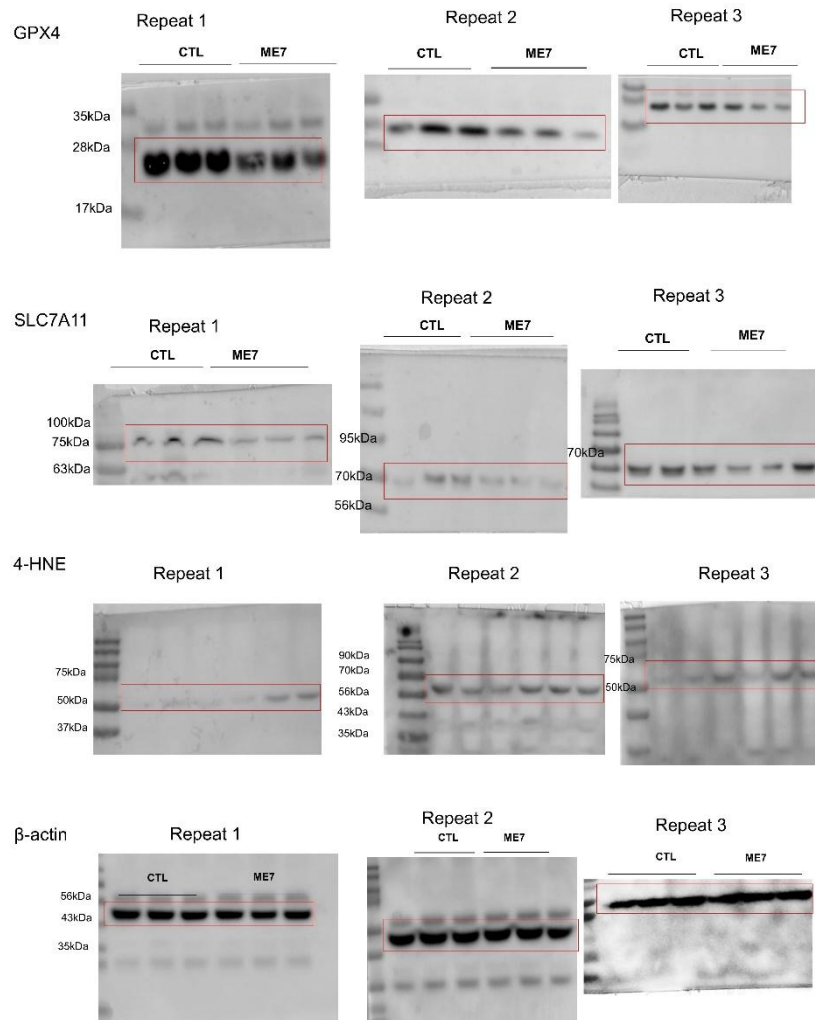

Supplement: Multimedia component 3 [file mmc3.pdf]
